# Defective Spermatogenesis: Martin et al. Respond

**DOI:** 10.1289/ehp.11489R

**Published:** 2008-08

**Authors:** Olwenn V. Martin, Tassos Shialis, Alan R. Boobis, Nikolaos Voulvoulis, John N. Lester, Mark D. Scrimshaw

**Affiliations:** Imperial College London, London, United Kingdom, E-mail: n.voulvoulis@imperial.ac.uk; Centre for Water Sciences, Cranfield University, Cranfield, United Kingdom; Institute for the Environment, Brunel University, Uxbridge, United Kingdom

In response to Prasad’s constructive comments on our quantitative meta-analysis of the estrogen hypothesis and testicular dysgenesis syndrome (Martin et al. 2008), we offer the following observations regarding the scope of our study and limitations of the methodology applied.

The primary objective of a quantitative meta-analysis is to combine the results of previous studies examining a specific research question to arrive at a summary conclusion about a body of research. This statistical pooling of several studies, taking into account the size of individual studies, confers more power to detect a potential association, and quantitative meta-analyses are often put at the top of evidence hierarchies. It cannot, however, correct for potential bias and confounding of the studies included; we addressed this issue in our review (Martin et al. 2008) by rating the quality of individual studies and carrying out a sensitivity analysis by excluding studies for which the quality score was below a chosen value. The method also requires that included studies report a measure of association such as a risk ratio or odds ratio. For this reason—although we did mention impaired spermatogenesis as one of the end points encompassed by the testicular dysgenesis syndrome—it was necessary to exclude this end point from our analysis.

In previous work and a scoping study, we found that most of the research carried out in relation to impaired spermatogenesis had investigated time trends rather than association with specific risk factors (Martin et al. 2007). Further, our analysis was limited to prenatal exposure to estrogenic agents. A number of studies have found associations between sperm motility or sperm DNA damage with levels of estrogenic chemicals measured either in urine or serum (Duty et al. 2003; Spanò et al. 2005). It would not be possible however to relate such levels to prenatal exposure. This also illustrates the difficulty of selecting a suitable marker of impaired spermatotogenesis.

Our study was implicitly limited to congenital cryptorchidism because the literature search did not yield any case–control or cohort studies that addressed the question of prenatal exposure to estrogenic compounds and acquired cryptorchidism in humans. In retrospect, this should have been explicitly stated in our article (Martin et al. 2008).

We concluded that the significant association between prenatal diethylstilbestrol exposure and all three end points considered conferred weight to the hypothesis of a common etiology for these disorders, and therefore to the existence of a testicular dysgenesis syndrome (Martin et al. 2008). Separate analyses were carried out for the three end points but the methodology applied did not allow us to explore the specific nature of causal relationships between congenital cryptorchidism, hypospadias, and testicular cancer. We are therefore grateful for Prasad’s insights.
